# Update cognition of nonalcoholic fatty liver disease/metabolism‐associated fatty liver disease

**DOI:** 10.1002/cdt3.14

**Published:** 2022-03-02

**Authors:** Weijun Gu, Yiming Mu

**Affiliations:** ^1^ Department of Endocrinology The First Medical Center of PLA General Hospital Beijing 100853 China

**Keywords:** metabolism associated fatty liver disease, nonalcoholic fatty liver disease

Nonalcoholic fatty liver disease (NAFLD) is a clinicopathological syndrome characterized by hepatic steatosis, excluding alcohol and other clear liver damage factors, including NAFL, nonalcoholic steatohepatitis (NASH), liver fibrosis, liver cirrhosis, and even liver cancer. NAFLD can progress from NAFL to NASH and then to cirrhosis, liver cancer,^1^ end‐stage liver disease, or even liver failure.^2^ NAFLD has become the most common cause of chronic liver disease, an emerging public health problem with an increasing prevalence in China. The global prevalence of NAFLD is as high as 25%. The prevalence of NAFLD in China is 29.2% and continues to rise. With a deeper understanding of the disease, increasing evidence has shown that NAFLD is a group of highly heterogeneous diseases, which is closely related to metabolic dysfunctions such as insulin resistance (IR), central obesity, dyslipidemia, hypertension, and hyperglycemia. It is the manifestation of metabolic syndrome in the liver.^3^ In 2020, more than 30 experts from 22 countries officially issued an international expert consensus statement to change the name of NAFLD to metabolism‐associated fatty liver disease (MAFLD).^4^ Whether to drink alcohol is no longer mentioned in the definition of MAFLD to avoid the possible diagnostic contradiction when alcoholic liver disease and MAFLD coexist. The new nomenclature highlights the central position of metabolic factors leading to liver fat deposition in MAFLD.

Since the prevalence of NAFLD and the mechanism of NAFLD progression are unclear, it is very important to clarify the potential mechanism of NAFLD in detail. According to the definition of “metabolism”, the latest name of the disease suggests that environmental factors are more important than genetic determinants. However, the interreaction of environmental and genetic factors that promote liver disease is unclear. The definition of MAFLD inevitably includes the presence of metabolite abnormalities. Although there is a possibility that environmental and genetic factors contribute to MAFLD, there is an orphan scenario that cannot be included in the scope of MAFLD, which is genetically acquired fatty liver disease (GAFLD) and hereditary hepatic steatosis without metabolic correlation. Fat accumulation, prevalence of NASH, and progression to advanced fibrosis are more likely to occur in patients with PNPLA3I148M gene mutation if there is no more severe IR.^5^ In addition, PNPLA3I148M gene variation plays an important role in lean patients with NAFLD with no Type 2 diabetes.^6^ This supports the concept that genetic variation may play a key role in lean NAFLD patients who may have GAFLD and do not meet the MAFLD criteria. Metwally et al. examined the relationship between copy number variations (CNVs) in exportin 4 (XPO4) with liver damage in MAFLD in a large cohort of patients and provided yet another proof that genetic variants may play an important role in some patients with hepatic steatosis.^7^ Only a few prior studies have examined the role of CNVs in the development or progression of NAFLD, and XPO4 CNVs have only been previously examined in an Asian population.

In recent years, many research teams in China have made significant contributions in mining hub genes or key genes and pathways of NAFLD using microarray technology; however, they remain unable to fundamentally elaborate the pathophysiological mechanism of the disease.^8^, ^9^, ^10^, ^11^, ^12^, ^13^ The present study integrated the available microarray datasets of human NAFLD liver tissues to perform comprehensive bioinformatic analysis of differentially expressed genes (DEGs), and hub genes were screened from a protein–protein interaction network and were verified using reverse real‐time‐quantitative polymerase chain reaction in a mouse model of NAFLD. This comprehensive analysis determined the candidate genes and pathways of NAFLD, as well as the DEGS and HUB genes associated with NAFLD in silico and in vitro. We identified 57 DEGs in mild and advanced NAFLD liver tissues. Nevertheless, further studies are required to clarify the detailed functions and specific mechanisms of these hub genes in the development and progression of NAFLD.

Based on these results, this genetic variant may not play a key role in the development of NAFLD. The answer may lie not only in one but in a combination of different genetic variants. The exact mechanism of NAFLD remains unclear, and there are no specific drugs for NAFLD. Therefore, innovative therapeutic strategies are required.

## CONFLICT OF INTERESTS

The authors declare no conflict of interest. Professor Yiming Mu is a member of Chronic Diseases and Translational Medicine editorial board and is not involved in the peer review process of this article.
